# Enhanced Cell Division Is Required for the Generation of Memory CD4 T Cells to Migrate Into Their Proper Location

**DOI:** 10.3389/fimmu.2019.03113

**Published:** 2020-01-15

**Authors:** Jana Sarkander, Shintaro Hojyo, Mathias Mursell, Yuzuru Yamasaki, Tsung-Yen Wu, Damon J. Tumes, Kosuke Miyauchi, Cam Loan Tran, Jinfang Zhu, Max Löhning, Andreas Hutloff, Mir-Farzin Mashreghi, Masato Kubo, Andreas Radbruch, Koji Tokoyoda

**Affiliations:** ^1^Deutsches Rheuma-Forschungszentrum Berlin, Leibniz Institute, Berlin, Germany; ^2^Centre for Cancer Biology, SA Pathology and The University of South Australia, Adelaide, SA, Australia; ^3^Laboratory for Cytokine Regulation, RIKEN Center for Integrative Medical Sciences, Yokohama, Japan; ^4^Laboratory of Immune System Biology, National Institute of Allergy and Infectious Diseases, National Institutes of Health, Bethesda, MD, United States; ^5^Experimental Immunology and Osteoarthritis Research, Department of Rheumatology and Clinical Immunology, Charité-Universitätsmedizin Berlin, Berlin, Germany; ^6^Division of Molecular Pathology, Research Institute for Biomedical Sciences, Tokyo University of Science, Noda, Japan

**Keywords:** CD4 T cells, memory, bone marrow, cell division, migration

## Abstract

CD4 T cell memory is fundamental for long-lasting immunity and effective secondary responses following infection or vaccination. We have previously found that memory CD4 T cells specific for systemic antigens preferentially reside in the bone marrow (BM) and arise from splenic CD49b^+^T-bet^+^ CD4 T cells. However, how BM-homing memory precursors are generated during an immune reaction is unknown. We show here that BM memory precursors are generated via augmented rates of cell division throughout a primary immune response. Treatment with the cytostatic drug cyclophosphamide or blockade of the CD28/B7 co-stimulatory pathway at the beginning of the contraction phase abrogates the generation of BM memory precursors. We determine that, following a critical number of cell divisions, memory precursors downregulate CCR7 and upregulate IL-2Rβ, indicating that loss of CCR7 and gain of IL-2 signal are required for the migration of memory precursors toward the BM.

## Introduction

Memory CD4 T cells are crucial for effective and long-lasting immunity against pathogens. During a primary immune response, CD4 T cells are activated by antigen-presenting cells (APCs), and following a period of clonal expansion, a small number of antigen-specific CD4 T cells differentiate into long-lived memory cells. The activation commonly occurs via interaction of the TCR and the peptide-MHC class II (pMHC) complex in secondary lymphoid organs (SLOs). To generate memory cells, it has been reported that the affinity and duration of interaction between the TCR and pMHC are critical, although co-stimulatory molecules, the cytokine milieu and the cellular microenvironment are also crucial determinants (1–3). High-affinity T cells differentiate into a large number of memory cells due to their greater expansion (4, 5). By contrast, in case of infection or lymphopenia, even very low-affinity antigens support memory development (6, 7). Regarding the duration of antigenic stimulation, Kim et al. have shown that Th1 memory fate correlates with longer duration of TCR-pMHC interaction, but not with the affinity of the TCR-pMHC interaction or the ability to expand (8). By contrast, Blair *et al*. have suggested that a lengthened period of antigenic stimulation during the primary immune response may be deleterious to memory generation (9). However, *in vitro*-differentiated effector cells could differentiate into memory cells in MHC class II-deficient hosts (10). Despite these many studies, it is still controversial how long antigen-specific naive CD4 T cells interact with APCs and how many rounds of cell division are required for the generation of memory cells *in vivo*. Previous studies in mice (11) and humans (12) revealed that resting memory CD4 T cells specific for systemic antigens preferentially reside in the BM, expressing Ly-6C highly. Moreover, BM memory CD4 T cells could produce cytokines more quickly *in vitro* and provide more efficient help for B cells *in vivo* compared to splenic cells (11). In mice, some activated CD4 T cells favorably migrate into the BM in an integrin α2 (CD49b)- and CD69-dependent manner during a primary immune response (11, 13, 14). We have previously defined CD49b^+^T-bet^+^ activated CD4 T cells generated during a primary immune response in the spleen as the precursors of BM memory CD4 T cells (15). However, it remains unclear how the duration of T cell-APC interaction and the rate of cell division upon activation influence the generation of BM memory precursors. We herein show that activated CD4 T cells require a specific amount of cell divisions to differentiate into BM memory precursors. In addition, the expression of the chemokine receptor CCR7 on the memory precursors is specifically downregulated with progressing rounds of cell division, whereas IL-2Rβ is upregulated, suggesting that the downregulation of CCR7 and the upregulation of IL-2Rβ in the course of enhanced cell division are required for the generation of BM memory CD4 T cells.

## Materials and Methods

### Mice

C57BL/6 (Charles River), Ly5.1 C57BL/6 (The Jackson Laboratory), Rag1-deficient (The Jackson Laboratory), ovalbumin-specific TCR transgenic (tg) (OT2, The Jackson Laboratory) (housed by DRFZ), lymphocytic choriomeningitis virus (LCMV) glycoprotein (GP)_61−80_-specific TCR tg (SMARTA) (16), and T-bet-ZsGreen reporter (17) mice were used. CCR7 KO mice were generated by the targeted deletion of the first part of exon 1 of the Ccr7 gene through co-injection of Cas9 mRNA and small guide RNAs for the target sequence (cagccaagccatgtaccttg) into C57BL/6 embryos. To examine the frequency of cycling cells, mice were injected intraperitoneally with 1 mg bromodeoxyuridine (BrdU) in 200 μl PBS once on day 5 after immunization. To block cell division and co-stimulatory signal, mice were treated with 50 mg/kg cyclophosphamide (CyP, Sigma) intravenously or 200 μg CTLA-4 Ig (BioXcell) intraperitoneally. To block CCL21 and IL-2, mice were treated with 50 μg of anti-CCL21 (goat IgG, R&D Systems) or 1 mg anti-IL-2 (S4B6) intraperitoneally. In each experiment, experimental groups were sex-matched and age-matched and used within 6–16 weeks of age. All mice were maintained under specific pathogen-free conditions. All mouse experiments were performed in accordance with the German law for animal protection and with permission from the responsible governmental authority, and in compliance with the guidelines of the Institutional Animal Care and Use Committee.

### Flow Cytometry

Flow cytometric analyses were conducted according to published guidelines (18). Single-cell suspensions were prepared from the spleen and BM of individual mice. The viability of cells was assessed by trypan blue exclusion. For cell surface staining, cells were stained for 20 min at 4°C with monoclonal antibodies against CD4 (GK1.5), CD122 (TM-β1), CD44 (IM7), B220 (RA3-6B2), CD49b (HMa2), CXCR3 (CXCR3-173), CD62L (MEL-14), NK1.1 (PK136), Thy1.1 (OX-7), CCR7 (4B12, BioLegend) PD-1 (HA2-7B1, Miltenyi) and respective isotype controls. PE-labeled MHC class II tetramers (I-A(b) LCMV GP_66−77_ and I-A(b) human CLIP_87−101_) were obtained through the NIH Tetramer Core Facility and stained as described (19). To exclude dead cells, cells were stained with 1 μg/ml propidium iodide prior to data acquisition (Sigma). Intracellular staining for Ki-67 (SolA15, ThermoFisher) and BrdU (BU20A, BioLegend) was performed according to the manufacturer's protocol. Cells were acquired on a BD Fortessa flow cytometer (BD Bioscience) and analyzed using the FlowJo X software (FlowJo, LLC).

### Cell Sorting, Adoptive Transfer, and Immunization

Naïve Thy1.1^+^ LCMV GP_61−80_-specific (SMARTA, LCMV GP-specific) CD4 T cells were negatively sorted with MojoSort™ Mouse CD4 Naïve T cell Isolation Kit (BioLegend) according to the manufacturer's protocol. To sort CFSE^hi/lo^ cells, a FACSAria cell sorter (BD Biosciences) was used. One million sorted naïve CD4 T cells were transferred intravenously. At the indicated time points mice were immunized intraperitoneally with 100 μg LCMV-GP_61−80_ peptide (LCMV-gp61, synthesized by Genecust) and 10 μg LPS (O111:B4, Sigma) in 200 μl PBS. To test cell division, naïve Thy1.1^+^ LCMV GP-specific CD4 T cells were labeled with 5 μM CFSE according to the manufacturer's protocol (BioLegend) followed by adoptive transfer and immunization.

### Immunofluorescent Staining and Confocal Microscopy

For immunofluorescence staining, samples were fixed in 4% paraformaldehyde overnight and equilibrated in 30% sucrose. Cryostat sections of adult spleen were stained with monoclonal antibodies against CD4 (RM4-5), Thy1.1 (HIS51) and CD11c (N418) (BioLegend). All histological analyses were carried out with a confocal laser microscope (LSM710, Carl Zeiss).

### Retroviral Infection

Viral particles were generated in HEK293T cells by calcium phosphate transfection using the packaging plasmid pCGP and the envelope plasmid pECO and (20, 21). A pQCXIX CD122 shRNA vector was generated by cloning the following oligonucleotides (forward 5′- TGGACCTCCTTGACATAAATTTCAAGAGAATTTATGTCAAGGAGGTCCTTTTTTC−3′, reverse 5′- TCGAGAAAAAAGGACCTCCTTGACATAAATTCTCTTGAAATTTATGTCAAGGAGGTCCA−3′) into the HpaI and XhoI restriction sites of pQCXIX. Splenocytes from Thy1.1^+^ SMARTA mice were stimulated with LCMV-gp61 for 24 h and then sorted CD4 T cells were added to virus-containing medium supplemented with HEPES-buffer (20 mM) and polybrene (8 μg/ml) and were centrifuged for 1.5 h at 32°C, 400 x g. As a control, a scrambled shRNA vector was constructed as described (22). pQCXIX has a GFP marker for positive gating.

### *In vitro* Stimulation of Naive CD4 T Cells

Naive CD4 T cells from C57BL/6 mice were isolated as described above and stimulated with plate-coated anti-CD3 (145-2C11, 1 μg/ml) and anti-CD28 (37–51, 10 μg/ml) in the presence of anti-IL-2 (S4B6, 20 μg/ml) or isotype control, in case of inhibitors, in the presence of Wortmannin (0.8 μM, Sigma), STAT5 inhibitor (20 μM, Tocris) or DMSO for 48 h at 37°C. Following stimulation, cells were washed and then incubated for 48 h.

### Statistical Analyses

All statistical analyses were performed using two-tailed Student's *t*-test.

## Results

### CD49b^±^CXCR3^±^ Memory Precursors Are Characterized by Enhanced Cell Division Throughout the Primary Immune Response

**Figure 1:**
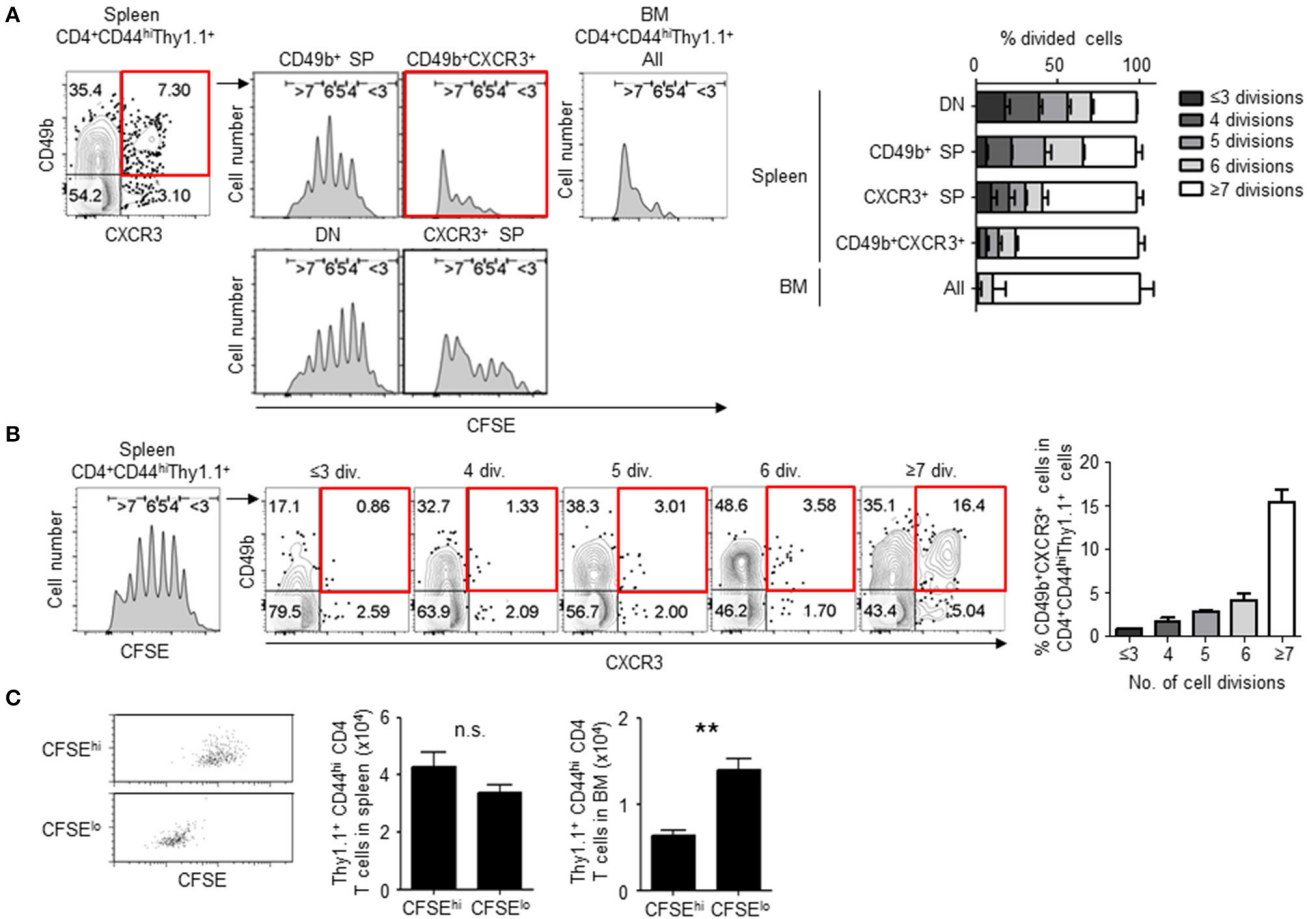
CD49b^±^CXCR3^±^ memory precursors exhibit enhanced cell division. Purified CFSE-labeled Thy1.1^+^ LCMV GP-specific CD4 T cells were transferred intravenously into C57BL/6 mice followed by intraperitoneal immunization with 100 μg LCMV-gp61 and 10 μg LPS, and analyzed on day 5 after immunization by flow cytometry. **(A)** Numbers of cell divisions in activated CD4 T cell subpopulations. A gating plot displays subsets defined by expression of CD49b and CXCR3 within Thy1.1^+^CD4^+^CD44^hi^B220^−^NK1.1^−^PI^−^ cells in the spleen and all subpopulations in the BM. Histograms display the dilution of CFSE in each subpopulations. Gates in histograms indicate the rounds of divisions that CFSE-labeled cells have undergone by day 5. A bar chart represents the percentages of each CFSE-diluted population *n* = 6. **(B)** Frequencies of the CD49b^+^CXCR3^+^ subpopulation in splenic activated CD4 T cells that have undergone the indicated numbers of cell divisions (div.) *n* = 6. **(C)** Highly divided CD4 T cells preferentially migrate into the BM. CFSE^hi^ and CFSE^lo^ cells were sorted from activated CD4 T cells on day 5 after immunization as described above, transferred into RAG1-deficient mice and 2 h later analyzed for Thy1.1^+^ cell numbers in the spleen and BM of host mice. The transfer efficiency was compensated by co-transferred Ly5.1^+^ splenocytes. Dot plots show the dilution of CFSE after sorting *n* = 4. These data are representative of two or more independent experiments. Data are shown as mean ± SD. **p < 0.01, n.s.: not significant. SP, single positive; DN, double negative.

We first examined the frequency of cell division in activated CD4 T cell subpopulations including BM memory precursors. We have previously found that splenic CD49b^+^T-bet^+^ CD4 T cells contain a population of BM memory precursors (15). T-bet directly trans-activates the gene encoding the chemokine receptor CXCR3 (23, 24). Hence, we hypothesized that CXCR3 would mirror T-bet expression and that CXCR3 would be expressed on the BM memory precursor subset. To test this, naive CD4 T cells from the spleen of T-bet-ZsGreen and LCMV GP-specific TCR tg mice were sorted and transferred them into C57BL/6 mice followed by immunization with LCMV-gp61 plus lipopolysaccharide (LPS). The expression of T-bet and CXCR3 in activated CD4 T cells directly correlated in the spleen and BM (Figure S1A) and most CD49b^+^T-bet^+^ (ZsGreen^+^) activated CD4 T cells in the spleen expressed CXCR3 (Figure S1B). Moreover, CXCR3^+^ activated CD4 T cells contained memory precursors as well as T-bet^+^ cells, because they preferentially migrated into the BM following cell transfer (Figure S1C). These data indicate that CXCR3 can be used as an equivalent marker to T-bet for the detection of memory precursors during a primary immune response. CFSE-labeled LCMV GP-specific naive CD4 T cells were transferred and four subpopulations defined by expression of CD49b and CXCR3 in LCMV GP-specific activated CD4 T cells were analyzed for cell division by CFSE dilution on day 5 after immunization, when CFSE fluorescence in divided cells was still detectable above background (Figure 1A). About 75% of the CD49b^+^CXCR3^+^ subpopulation had divided 7 or more times (≥7) compared to other subpopulations that ranged from 28 to 58% of cells that had divided more than 7 times (Figure 1A). Alternatively, in an immune response to ovalbumin, CD49b^+^CXCR3^+^ subpopulation of the antigen-specific activated CD4 T cells also divided more times compared to other subpopulations (Figure S1D). In line with this, the CD49b^+^CXCR3^+^ subpopulation was strongly enriched in the most divided (≥7) LCMV GP-specific activated CD4 T cells, increasing from 3.5% of cells to 16.4% of cells after the 6th division (Figure 1B). Interestingly, about 90% of cells that had already migrated into the BM have experienced ≥7 rounds of cell division (Figure 1A). To examine whether cell division is associated with the ability to migrate into the BM, CFSE^hi^ and CFSE^lo^ activated CD4 T cells were sorted from the spleen on day5 after immunization and transferred into naive mice (Figure 1C). Significantly, CFSE^lo^ cells were enriched in the BM compared to CFSE^hi^ cells, suggesting that highly divided activated CD4 T cells contain BM memory precursors. In chronic immune response, some highly divided CD8 T cells can be exhausted, expressing PD-1 (25). Although BM memory CD4 T cells express PD-1 lowly (Figure S1E), they are sufficiently functional *in vivo* and *in vitro* (11, 19).

**Figure 2:**
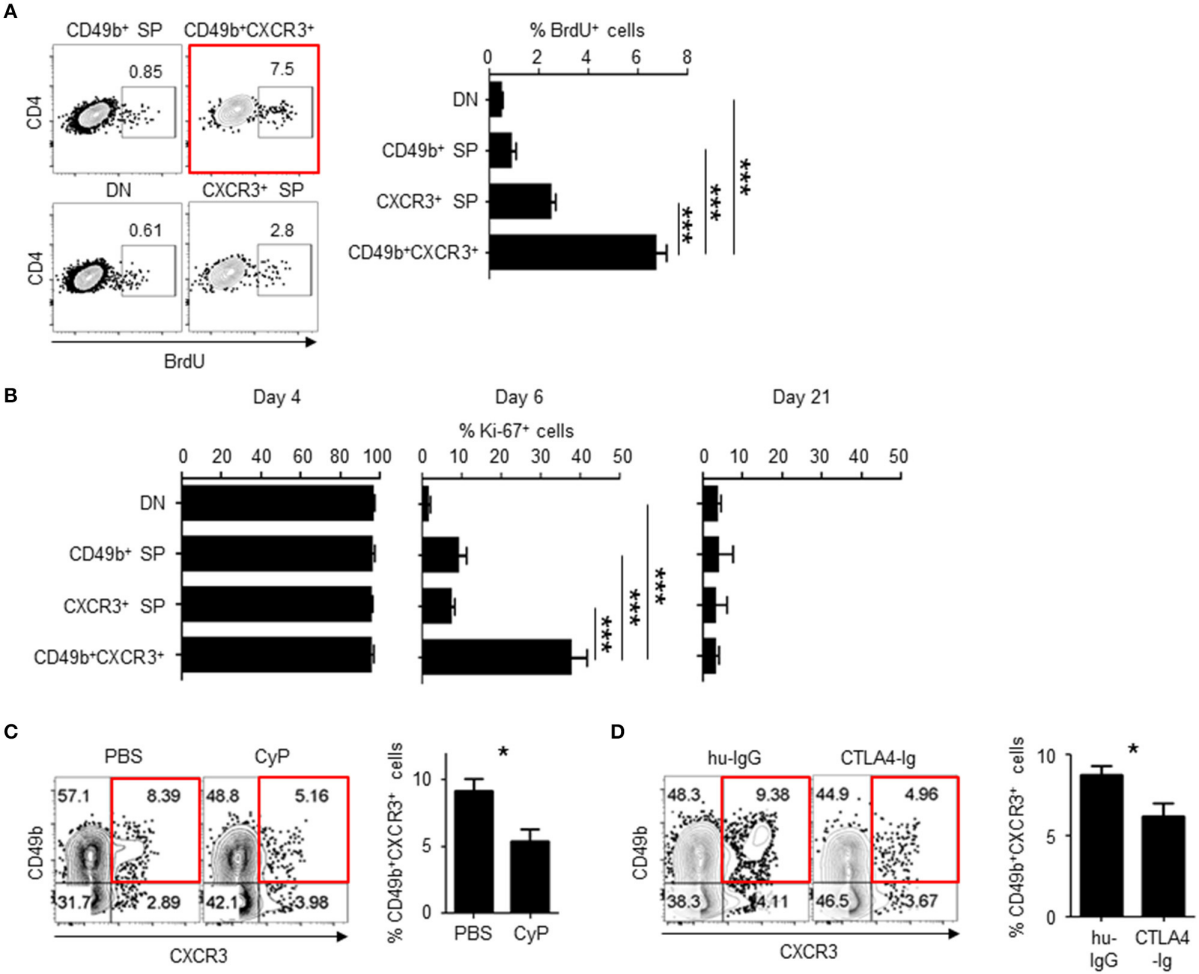
CD49b^±^CXCR3^±^ memory precursors are highly dividing throughout the primary immune response. C57BL/6 mice were transferred with purified Thy1.1^+^ LCMV GP-specific CD4 T cells, immunized with LCMV-gp61 and LPS and on day 6 after immunization analyzed by flow cytometry and confocal microscopy. **(A)** More CD49b^+^CXCR3^+^ memory precursors incorporated BrdU at the beginning of the contraction phase of a primary immune response. Mice were injected intraperitoneally with 1 mg of BrdU on day 5 after immunization. Dot plots show the expression of BrdU in each subpopulation (right) and a bar chart shows the percentage of BrdU^+^ cells in each subpopulation *n* = 9. **(B)** Expression of Ki-67 in each subpopulation of activated CD4 T cells. A bar chart represents the percentages of Ki-67^+^ cells in splenic subpopulations defined by the expression of CD49b and CXCR3 on days 4, 6, and 21 after immunization *n* = 7. **(C)** Injection of CyP reduces numbers of BM memory precursors. Mice were injected intravenously with 50 mg/kg CyP or PBS on day 5 after immunization. Dot plots show the expression of CD49b and CXCR3 within splenic Thy1.1^+^CD44^hi^CD4^+^B220^−^NK1.1^−^PI^−^ cells of CyP- or PBS-treated mice. A bar chart displays the percentages of the CD49b^+^CXCR3^+^ subpopulation *n* = 11. **(D)** CTLA-4 Ig reduces numbers of BM memory precursors. Mice were injected intraperitoneally with 200 μg CTLA-4 Ig or human IgG on days 4 and 5 after immunization *n* = 12. These data are representative of two or more independent experiments. Data are shown as mean ± SD. *p< 0.05, ***p< 0.001.

In our immunization model with antigen plus LPS, antigen-specific CD4 T cells in the SLOs expand until day 4 after immunization and then quickly decrease in the contraction phase of the immune response (11, 13). To examine if cell division of CD49b^+^CXCR3^+^ activated CD4 T cells is sustained at the beginning of the contraction phase of a primary immune response (i.e., on days 5 and 6 after immunization), LCMV GP-specific CD4 T cell-transferred mice were injected with BrdU on day 5 after immunization and analyzed for the incorporation of BrdU in four subpopulations defined by different expression of CD49b and CXCR3 on day 6 after immunization (Figure 2A). BrdU^+^ cycling LCMV GP-specific CD4 T cells were significantly enriched within the CD49b^+^CXCR3^+^ subpopulation. Ki-67 is a marker strictly associated with cell proliferation, which is present during all active phases of the cell cycle (G_1_, S, G_2_ and mitosis) but is absent in resting (quiescent) cells (G_0_) (26). In all subpopulations, the expression of Ki-67 was not different on days 4 and 21 after immunization (Figure 2B, left and right). Remarkably, a much larger proportion (38%) of the CD49b^+^CXCR3^+^ subpopulation expressed Ki-67 on day 6 after immunization, compared to 1–9% of Ki-67^+^ cells within the other subpopulations (Figure 2B, middle). These data suggest that BM memory precursors undergo sustained cell division for longer times than the non-CD49b^+^CXCR3^+^ subpopulations.

### Expansion of CD49b^±^CXCR3^±^ Activated CD4 T Cells Can Be Diminished by Inhibition of Cell Proliferation and Co-stimulatory Signals in the Contraction Phase of a Primary Immune Response

**Figure 3:**
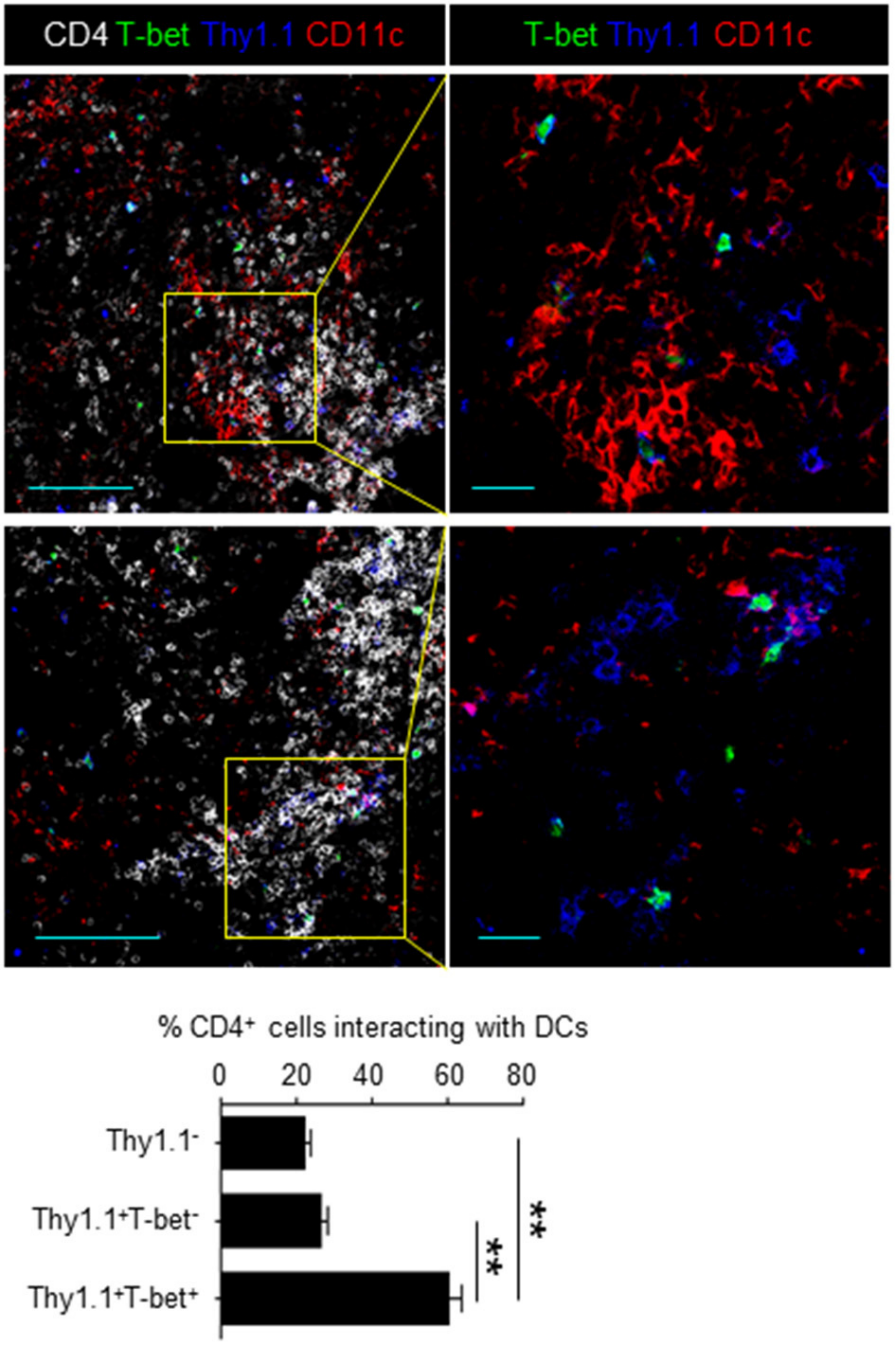
Memory precursors preferentially contact DCs at the beginning of the contraction phase. Splenic sections from mice transferred with T-bet-ZsGreen (green) tg LCMV GP-specific CD4 T cells and immunized were stained with anti-Thy1.1 (blue), anti-CD11c (red) and anti-CD4 (white). A bar chart represents the quantification of Thy1.1^−^ (endogenous CD4^+^), T-bet^−^Thy1.1^+^ and T-bet^+^Thy1.1^+^ cells in contact with CD11c^+^ cells in the PALS. Scale bar, 100 μm (left) and 20 μm (right) *n* = 100–200. These data are representative of three independent experiments. Data are shown as mean ± SD. **p < 0.01.

Cell proliferation can be abrogated with a cytostatic drug, cyclophosphamide (CyP) (27). To demonstrate that CD49b^+^CXCR3^+^ activated CD4 T cells are affected by treatment with CyP at the beginning of the contraction phase, mice were transferred with LCMV GP-specific naive CD4 T cells, injected with CyP or PBS on day 5 after immunization and analyzed for the expression of CD49b and CXCR3 in splenic LCMV GP-specific CD4 T cells on day 6 after immunization (Figure 2C). The frequency of the CD49b^+^CXCR3^+^ subpopulation was significantly reduced by treatment with CyP. Co-stimulatory signaling via the interaction of CD28 expressed on T cells with B7 expressed on APCs is essential for the functional activation and proliferation of antigen-specific CD4 T cells. To examine whether co-stimulation signaling at the beginning of the contraction phase is involved in the expansion of highly proliferative CD49b^+^CXCR3^+^ activated CD4 T cells, the CD28/B7 co-stimulatory pathway was blocked by administration of a CTLA-4 Ig fusion protein (28). Transferred and immunized mice were injected with CTLA-4 Ig or a human IgG control (hu-IgG) on days 4 and 5 after immunization and analyzed on day 6 (Figure 2D). The frequencies of the CD49b^+^CXCR3^+^ subpopulation within LCMV GP-specific CD4 T cells were significantly and specifically reduced in CTLA-4 Ig-treated animals compared to the hu-IgG treated controls. To test whether the sustained cell proliferation of the CD49b^+^CXCR3^+^ subpopulation is induced by prolonged contact with DCs, frozen sections of spleens harvested on day 6 after immunization were histologically analyzed for interaction of DCs with T-bet^+^ or T-bet^−^ Thy1.1^+^ antigen-specific CD4 T cells or Thy1.1^−^ endogenous CD4 T cells. Around 60% of T-bet^+^ antigen-specific CD4 T cells were found to be in contact with CD11c^+^ DCs in the T cell area, called the periarteriolar lymphoid sheaths (PALS) of the spleen, whereas only 25% of T-bet^−^ cells contacted CD11c^+^ cells (Figure 3). These data indicate that CD49b^+^CXCR3^+^ activated CD4 T cells require sustained cell proliferation and co-stimulatory signaling that extends into the contraction phase of a primary immune response.

### Highly Proliferative CD49b^±^CXCR3^±^ Activated CD4 T Cells Rapidly Downregulate CCR7 and Upregulate IL-2Rβ

**Figure 4:**
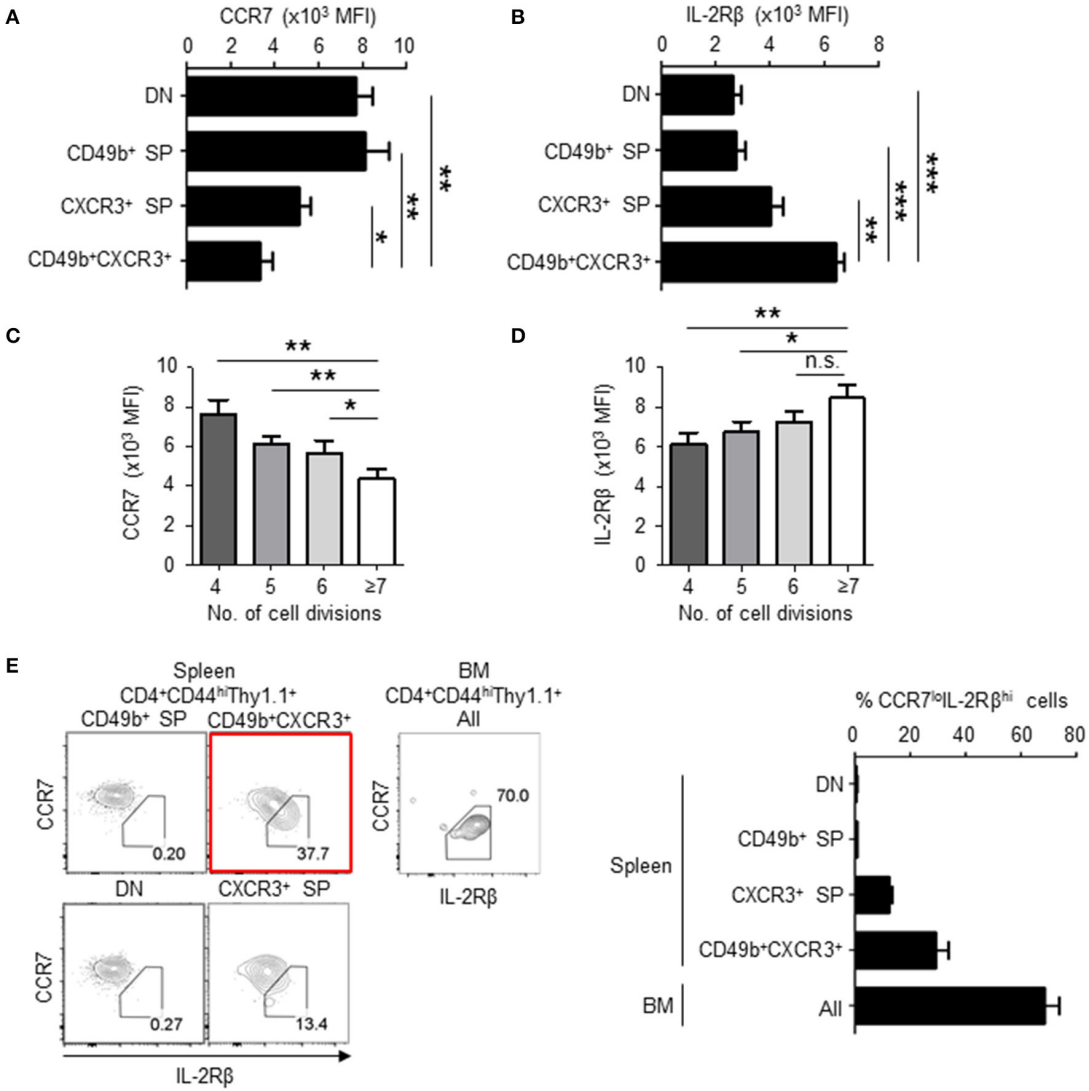
BM memory precursors downregulate CCR7 and upregulate IL-2Rβ following cell division. As described in Figure 1, C57BL/6 mice were transferred, immunized and analyzed by flow cytometry on day 5 after immunization. **(A,B)** CD49b^+^CXCR3^+^ memory precursors express low level of CCR7 and high level of IL-2Rβ. The geometric mean of fluorescent intensity (MFI) of CCR7 **(A)** and IL-2Rβ **(B)** expression in splenic subpopulations defined by expression of CD49b and CXCR3 is shown *n* = 8. **(C,D)** Cells with enhanced numbers of divisions downregulate CCR7 and upregulate IL-2Rβ in the CD49b^+^CXCR3^+^ subpopulation. The GMFI of CCR7 **(C)** and IL-2Rβ **(D)** within the CD49b^+^CXCR3^+^ subpopulation is shown for each cell division (*n* = 7). **(E)** IL-2Rβ^hi^CCR7^lo^ cells are enriched in the CD49b^+^CXCR3^+^ subpopulation. Gating plots (left) show the expression of CCR7 and IL-2Rβ in splenic subpopulations defined by expression of CD49b and CXCR3 and all subpopulations of the BM. A bar chart displays the percentages of IL-2Rβ^hi^CCR7^lo^ cells in each subpopulation of the spleen and all subpopulations of the BM *n* = 8. These data are representative of two independent experiments. Data are shown as mean ± SD. *p < 0.05, **p < 0.01, ***p < 0.001, n.s.: not significant.

We have previously shown that BM resting memory CD4 T cells express low levels of CCR7 and high levels of IL-2Rβ (11), and that BM memory precursors relocate from the PALS to the red pulp around day 6 after immunization (15). CCR7 contributes to the migration and persistence of CD4 T cells in(to) the PALS of the spleen (29) and IL-2 signaling is required for the transition of effector to memory CD4 T cells (30). On day 6 after immunization, CD49b^+^CXCR3^+^ activated CD4 T cells significantly downregulated CCR7 and upregulated IL-2Rβ compared to other subpopulations (Figures 4A,B). Expression of IL-2Rα was already lost in all the subpopulations on days 4 and 6 (data not shown). Downregulation of CCR7 and upregulation of IL-2Rβ in CD49b^+^CXCR3^+^ activated CD4 T cells also correlated with the number of cell divisions (Figures 4C,D). Our data indicate that downregulation of CCR7 and upregulation of IL-2Rβ first occurs in the spleen, and that the majority of BM memory CD4 T cells are IL-2Rβ^hi^CCR7^lo^. The splenic precursors of BM CD4 memory T cells can therefore be defined as CD49b^+^CXCR3^+^IL-2Rβ^hi^CCR7^lo^ cells (Figure 4E).

### Highly Proliferative CD49b^±^CXCR3^±^ Activated CD4 T Cells Are Defined by Loss of CCR7 and Gain of IL-2Rβ

**Figure 5:**
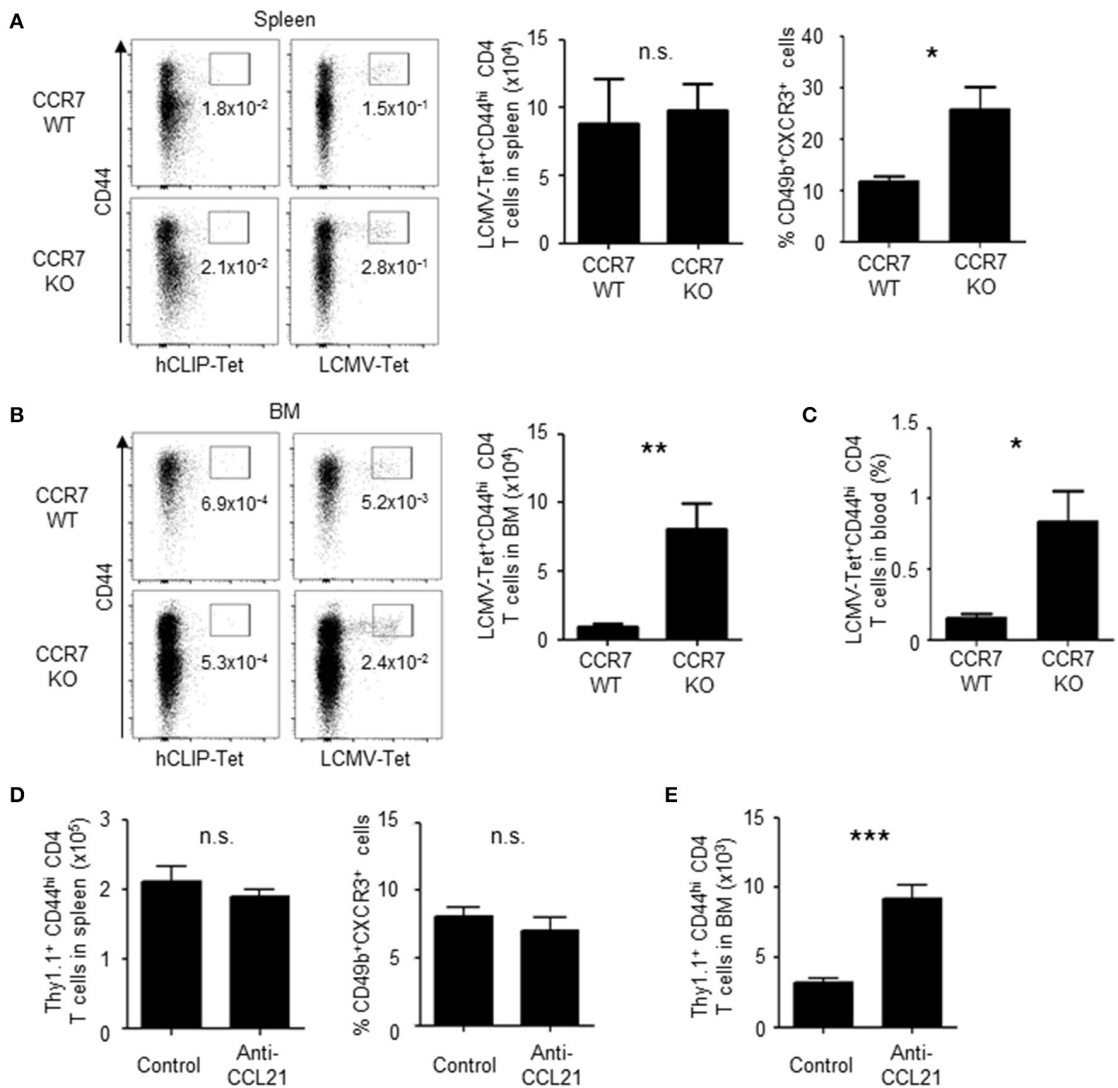
Loss of CCR7 enhances the differentiation of BM memory precursors and the migration of activated CD4 T cells into the BM. **(A–C)** A deficiency of CCR7 increases the migration of memory precursors into the BM. CCR7 WT or KO mice were immunized twice with LCMV-gp61 and LPS and on day 6 after the second immunization analyzed for the enumeration of LCMV GP-specific CD4 T cells in the spleen **(A)**, BM **(B)** and peripheral blood **(C)** by LCMV GP-specific MHC class II tetramer staining. Dot plots show the expression of CD44 and binding of LCMV GP-specific tetramer or control tetramer (hCLIP) in CD4^+^B220^−^NK1.1^−^PI^−^ cells. Bar charts show the numbers and frequencies of LCMV-tetramer positive CD44^hi^ CD4 T cells **(A–C)** and the frequency of CD49b^+^CXCR3^+^ cells within splenic LCMV-tetramer positive CD44^hi^ CD4 T cells **(A)** *n* = 9. **(D,E)** Blockade of CCL21 increases the migration of memory precursors into the BM. Mice transferred and immunized as described in Figure 1 were injected intraperitoneally with 50 μg anti-CCL21 or isotype control on day 5 after immunization. Numbers of total Thy1.1^+^ CD4 T cells and the frequency of the CD49b^+^CXCR3^+^ subpopulation in the spleen **(D)** and numbers of total Thy1.1^+^ CD4 T cells in the BM **(E)** on day 6 after immunization are shown *n* = 4. These data are representative of two independent experiments. Data are shown as mean ± SD. *p < 0.05, **p < 0.01, ***p < 0.001, n.s.: not significant.

To determine if downregulation of CCR7 promotes the migration of memory precursors to the BM, antigen-specific CD4 T cells in CCR7 wildtype (WT) or knockout (KO) mice were analyzed on day 6 after immunization (Figures 5A–C). In these mice, antigen-specific CD4 T cells were detected by MHC class II tetramer staining for LCMV GP_66−77_. In the spleen, CCR7-deficient antigen-specific CD4 T cells were activated and expanded normally (Figure 5A). However, about 2-fold more CCR7-deficient memory precursors in the spleen, about 5-fold more in the blood and 8-fold more CCR7-deficient antigen-specific CD4 T cells in the BM, were detected compared to WT cells (Figures 5A–C), suggesting that the downregulation of CCR7 positively contributes to the migration of the memory precursors toward the BM. In addition, the increased migration into the BM was also evident when CCL21, a major ligand of CCR7 in the spleen (31–33), was blocked once on day 5 after immunization, although memory precursors in the spleen were not increased (Figures 5D,E).

**Figure 6:**
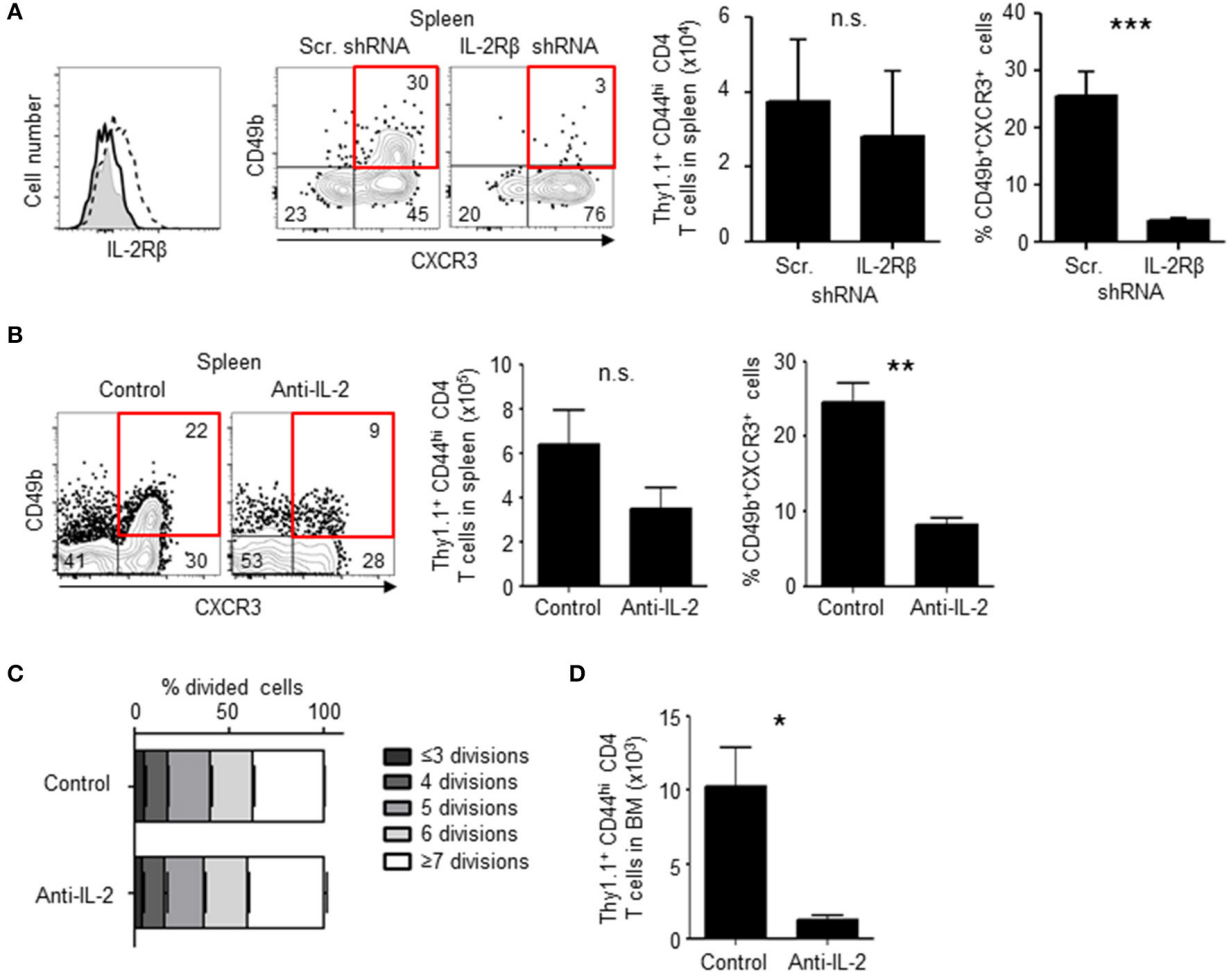
Loss of IL-2 signal inhibits the differentiation of BM memory precursors. **(A)** Knock-down of IL-2Rβ reduces numbers of CD49b^+^CXCR3^+^ memory precursors. C57BL/6 mice were transferred with preactivated and IL-2Rβ shRNA- or scrambled (Scr.) shRNA-transduced Thy1.1^+^ LCMV GP-specific CD4 T cells, immunized with LCMV-gp61 and LPS and analyzed on day 6 after immunization by flow cytometry. The histogram shows the efficiency of IL-2Rβ shRNA-mediated knock-down (filled histogram, isotype control; dashed line, Scr. shRNA; solid line, IL-2Rβ shRNA) and the dot plots show the expression of CD49b and CXCR3 in Thy1.1^+^CD44^hi^CD4^+^B220^−^NK1.1^−^PI^−^ cells. The bar charts display the numbers of total Thy1.1^+^ LCMV GP-specific CD4 T cells (left) and the frequency of CD49b^+^CXCR3^+^ subpopulation (right) *n* = 6. **(B–D)** Injection of anti-IL-2 reduces numbers of CD49b^+^CXCR3^+^ memory precursors. As described in Figure 1, C57BL/6 mice were transferred, immunized and analyzed by flow cytometry on day 6 after immunization, receiving 1 mg of anti-IL-2 or isotype control on days 0, 2, and 4. The dot plots show the expression of CD49b and CXCR3 in Thy1.1^+^CD44^hi^ CD4 T cells. The bar charts display the numbers of total Thy1.1^+^ CD4 T cells (**B**, left) and the frequency of CD49b^+^CXCR3^+^ subpopulation (**B**, right), the dilution of CFSE in each subpopulation in the spleen **(C)** and the BM **(D)** *n* = 4. These data are representative of two independent experiments. Data are shown as mean ± SD. *p < 0.05, **p < 0.01, ***p < 0.001, n.s.: not significant.

**Figure 7:**
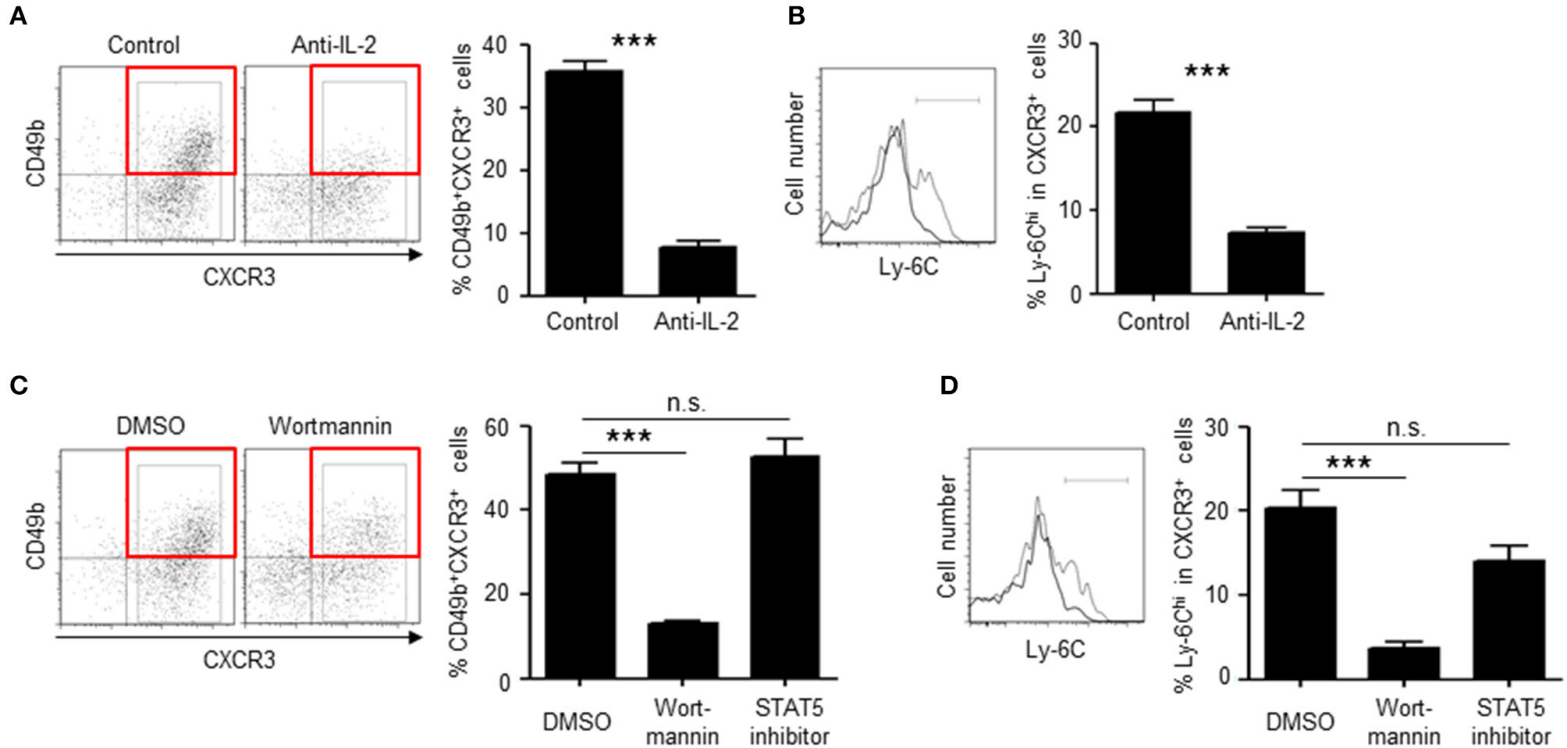
IL-2 enhances the differentiation of memory precursors via PI3K *in vitro*. **(A,B)** Blockade of IL-2 reduces the frequency of CD49b^+^CXCR3^+^ subpopulation in *in vitro*-activated CD4 T cells. Naive CD4 T cells from C57BL/6 mice were stimulated with anti-CD3/CD28 for 48 h in the presence of anti-IL-2 or isotype control and then incubated for 48 h. The frequencies of CD49b^+^CXCR3^+^ in activated CD4 T cells **(A)** and Ly-6C^hi^ cells in CXCR3^+^ activated CD4 T cells **(B)** are shown. **(C,D)** Inhibition of PI3K reduces the frequency of CD49b^+^CXCR3^+^ subpopulation in *in vitro*-activated CD4 T cells. Naive CD4 T cells from C57BL/6 mice were stimulated with anti-CD3/CD28 for 48 h in the presence of Wortmannin, STAT5 inhibitor or DMSO and then incubated for 48 h. The frequencies of CD49b^+^CXCR3^+^ in activated CD4 T cells **(C)** and Ly-6C^hi^ cells in CXCR3^+^ activated CD4 T cells **(D)** are shown *n* = 6. These data are representative of two independent experiments. Data are shown as mean ± SD. ***p < 0.001, n.s.: not significant.

To investigate the role of IL-2Rβ in the generation of BM memory CD4 T cells, the expression of IL-2Rβ was knocked-down by retrovirus-mediated RNA interference (Figure 6A). Knockdown of IL-2Rβ reduced the frequencies of CD49b^+^CXCR3^+^ CD4 T cells, in particular the expression of CD49b, a homing receptor of CD4 T cells to the BM (14), but not the expansion of antigen-specific CD4 T cells. Similarly, mice that were injected with anti-IL-2 antibodies during the activation phase exhibited an impaired generation of CD49b^+^CXCR3^+^ CD4 T cells in the spleen and an impaired migration of antigen-specific CD4 T cells into the BM while numbers and cell division of antigen-specific CD4 T cells in the spleen were not greatly affected (Figures 6B–D). To test whether loss of IL-2 signal directly affects the expression of CCR7, mice that were injected with anti-IL-2 antibodies were analyzed for the expression of CCR7 (Figure S1F). However, a blockade of IL-2 did not affect the expression of CCR7 in the remaining CD49b^−^ CD4 T cell population. Some naive CD4 T cells stimulated *in vitro* by anti-CD3 and anti-CD28 antibodies for 2 days and then incubated without stimulation for 2 additional days express CD49b and CXCR3 (34, 35). To test whether the co-expression of CD49b and CXCR3 is affected by IL-2 blockade, naive CD4 T cells were stimulated in the presence of anti-IL-2 or the isotype control. Treatment of anti-IL-2 reduced the frequencies of CD49b^+^CXCR3^+^ CD4 T cells and CD4 T cells highly expressing Ly-6C, a marker of BM-resident memory CD4 T cells (Figures 7A,B). IL-2 induces the activation of phosphoinositide 3-kinase (PI3K) and signal transducer and activator of transcription 5 (STAT5) (36). Inhibition of PI3K but not STAT5 reduced the frequencies of CD49b^+^CXCR3^+^ and Ly-6C^hi^ CD4 T cells (Figures 7C,D), suggesting that IL-2 enhance the generation of memory precursors via PI3K activation. These data show that the egress from the spleen and the migration into the BM is controlled by downregulation of CCR7 and upregulation of IL-2Rβ. In summary, our data indicate that the enhanced cell division leads to the transition of activated CD4 T cells into BM memory precursors.

## Discussion

We have previously shown that splenic CD49b^+^T-bet^+^ activated CD4 T cells include the precursors of resting BM memory CD4 T cells. We herein show that CD49b^+^CXCR3(T-bet)^+^ BM memory precursors are differentiated through enhanced proliferation that extends into the contraction phase of a primary immune response. Treatment with cyclophosphamide and CTLA-4 Ig after the peak of cell expansion specifically reduced the numbers of BM memory precursors. BM memory precursors that had divided 7 times or more, displayed the lowest expression of CCR7 and highest levels of IL-2Rβ. A deficiency of CCR7 or a blockade of CCL21 markedly increased the migration of memory precursors into the BM whereas a knockdown of IL-2Rβ and a blockade of IL-2 reduced the numbers of BM memory precursors by suppressing the expression of CD49b, a homing receptor of CD4 T cells to the BM. Our data therefore indicate that differentiation of BM memory precursors requires sustained cell division throughout a primary immune response to migrate toward the BM.

It has remained controversial whether memory CD4 T cells have differential requirements for cell division to obtain longevity. Chang et al. have previously suggested an asymmetric division model for effector and memory T cell differentiation (37). To become an effector cell, T cells need to contact APCs proximally and divide more. By contrast, T cells located distally to the APCs stop cell division earlier and are proposed to favor memory generation. However, we show here that BM memory precursors are in prolonged contact with DCs and divide more than other responding subpopulations. We have previously shown that B cells suppress the generation of BM memory CD4 T cells (15), and other groups have shown that B cells enhance the generation of splenic memory CD4 T cells (38–41). These data show that BM and splenic memory CD4 T cells are generated in a distinct manner, although it still remains unclear if splenic memory CD4 T cells are generated according to an asymmetric division model. Our study, rather, supports a long duration model of interaction with DCs for BM memory CD4 T cell formation, and suggests that enhanced cell division contributes to the migration toward the BM. We here show the formation of antigen-specific memory CD4 T cells in an immune response. Based on these findings, the formation of pathogen-specific memory CD4 T cells in a persistent immune response, e.g., LCMV infection, could be investigated.

CCR7 is required for the persistence of CD4 T cells in the T cell area of SLOs (29). We have previously shown that T-bet^+^ activated CD4 T cells preferentially egress from the T cell area of the spleen (15). To egress from the T cell area into the red pulp or other tissues, e.g., BM, memory precursors require the downregulation of CCR7 coupled with sufficient numbers of cell divisions. It still remains unclear, however, how cell division affects the transcription and chromatin remodeling of IL-2Rβ and CCR7. In addition, antigen-specific CD4 T cells may expand more in CCR7-deficient situation, since the sum of splenic and BM antigen-specific CD4 T cells increases twice in CCR7-deficient mice. These results suggest that in CCR7-deficient mice, CD4 T cells can grow well in the expanded area and also can efficiently generate memory cells by loss of CCR7. The transition of effector to memory cells has been described to be dependent on IL-2 (30). Additionally, IL-2 signaling during priming has been shown to enhance long-term survival of CD4 T cells (42). Thus, to survive long-term as memory cells, CD4 T cells require the early upregulation of IL-2Rβ. We here show that IL-2 signals via IL-2Rβ induce not only survival but also the PI3K-dependent expression of CD49b which is required for the transmigration of activated CD4 T cells into the BM through sinusoids (14). However, a link between the activation of PI3K and the transcription/translation of CD49b should be further investigated. In conclusion, we propose that prolonged DC contact and enhanced cell division direct the migration of memory precursors into BM survival niches via downregulation of CCR7 and the upregulation of IL-2Rβ.

## Data Availability Statement

The datasets generated for this study are available on request to the corresponding author.

## Ethics Statement

The animal study was reviewed and approved by the Landesamt für Gesundheit and Soziales, Berlin, Germany.

## Author Contributions

JS, MM, SH, YY, T-YW, KM, and CT conducted the experiments. JS and KT designed the experiments and wrote the paper. DT edited the paper. JZ, ML, AH, M-FM, and MK provided materials and tools. ML, AH, and AR supervised the study.

### Conflict of Interest

The authors declare that the research was conducted in the absence of any commercial or financial relationships that could be construed as a potential conflict of interest.

## References

[1] Swain SL Agrewala JN Brown DM Jelley-gibbs DM Golech S Huston G CD4^+^ T-cell memory: generation and multi-faceted roles for CD4^+^ T cells in protective immunity to influenza. Immunol Rev. (2008) 1:8–22. 10.1111/j.0105-2896.2006.00388.x PMC2266984 16824113

[2] Gasper DJ Tejera MM Suresh M. CD4 T-cell memory generation and maintenance. Crit Rev Immunol. (2014) 34:121–46. 10.1615/CritRevImmunol.2014010373 24940912 PMC4062920

[3] Arsenio J Metz PJ Chang JT. Asymmetric cell division in T lymphocyte fate diversification. Trends Immunol. (2015) 36:670–83. 10.1016/j.it.2015.09.004 26474675 PMC4640954

[4] Corse E Gottschalk RA Allison JP. Strength of TCR-peptide/MHC interactions and in vivo T cell responses. J Immunol. (2011) 186:5039–45. 10.4049/jimmunol.1003650 21505216

[5] Kuhns MS Davis MM. TCR signaling emerges from the sum of many parts. Front Immunol. (2012) 3:159. 10.3389/fimmu.2012.00159 22737151 PMC3381686

[6] Zehn D Lee SY Bevan MJ. Complete but curtailed T-cell response to very low-affinity antigen. Nature. (2009) 458:211–4. 10.1038/nature07657 19182777 PMC2735344

[7] Sabatino JJ Huang J Zhu C Evavold BD. High prevalence of low affinity peptide–MHC II tetramer-negative effectors during polyclonal CD4^+^ T cell responses. J Exp Med. (2011) 208:81–90. 10.1084/jem.20101574 21220453 PMC3023139

[8] Kim C Wilson T Fischer KF Williams MA. Sustained interactions between T cell receptors and antigens promote the differentiation of CD4^+^ memory T cells. Immunity. (2013) 39:508–20. 10.1016/j.immuni.2013.08.033 24054329 PMC3816772

[9] Blair DA Turner DL Bose TO Pham QM Bouchard KR Williams KJ. Duration of antigen availability influences the expansion and memory differentiation of T cells. J Immunol. (2011) 187:2310–21. 10.4049/jimmunol.1100363 21775679 PMC3159832

[10] Swain SL Hu H Huston G. Class II-independent generation of CD4 memory T cells from effectors. Science. (1999) 286:1381–3. 10.1126/science.286.5443.1381 10558997

[11] Tokoyoda K Zehentmeier S Hegazy AN Albrecht I Grün JR Löhning M. Professional memory CD4^+^ T lymphocytes preferentially reside and rest in the bone marrow. Immunity. (2009) 30:721–30. 10.1016/j.immuni.2009.03.015 19427242

[12] Okhrimenko A Grun JR Westendorf K Fang Z Reinke S von Roth P. Human memory T cells from the bone marrow are resting and maintain long-lasting systemic memory. Proc Natl Acad Sci USA. (2014) 111:9229–34. 10.1073/pnas.1318731111 24927527 PMC4078840

[13] Shinoda K Tokoyoda K Hanazawa A Hayashizaki K Zehentmeier S Hosokawa H. Type II membrane protein CD69 regulates the formation of resting T-helper memory. Proc Natl Acad Sci USA. (2012) 109:7409–14. 10.1073/pnas.1118539109 22474373 PMC3358871

[14] Hanazawa A Hayashizaki K Shinoda K Yagita H Okumura K Löhning M. CD49b-dependent establishment of T helper cell memory. Immunol Cell Biol. (2013) 91:524–31. 10.1038/icb.2013.36 23897120

[15] Hojyo S Sarkander J Manne C Mursell M Hanazawa A Zimmel D. B cells negatively regulate the establishment of CD49b(+)T-bet(+) resting memory T helper cells in the bone marrow. Front Immunol. (2016) 7:26. 10.3389/fimmu.2016.00026 26870041 PMC4735404

[16] Oxenius A Bachmann MF Zinkernagel RM Hengartner H. Virus-specific MHC-class II-restricted TCR-transgenic mice: effects on humoral and cellular immune responses after viral infection. Eur J Immunol. (1998) 28:390–400. 9485218 10.1002/(SICI)1521-4141(199801)28:01<390::AID-IMMU390>3.0.CO;2-O

[17] Zhu J Jankovic D Oler AJ Wei G Sharma S Hu G. The transcription factor T-bet is induced by multiple pathways and prevents an endogenous Th2 cell program during Th1 cell responses. Immunity. (2012) 37:660–73. 10.1016/j.immuni.2012.09.007 23041064 PMC3717271

[18] Cossarizza A Chang HD Radbruch A Andrä I Annunziato F Bacher P. Guidelines for the use of flow cytometry and cell sorting in immunological studies. Eur J Immunol. (2017) 47:1584–797. 10.1002/eji.201646632 29023707 PMC9165548

[19] Siracusa F McGrath MA Maschmeyer P Bardua M Lehmann K Heinz G. Nonfollicular reactivation of bone marrow resident memory CD4 T cells in immune clusters of the bone marrow. Proc Natl Acad Sci USA. (2018) 115:1334–9. 10.1073/pnas.1715618115 29358404 PMC5819416

[20] Weber JP Fuhrmann F Feist RK Lahmann A Al Baz MS Gentz LJ. ICOS maintains the T follicular helper cell phenotype by down-regulating Krüppel-like factor 2. J Exp Med. (2015) 212:217–33. 10.1084/jem.20141432 25646266 PMC4322049

[21] Haftmann C Stittrich AB Zimmermann J Fang Z Hradilkova K Bardua M. MiR-148a is upregulated by Twist1 and T-bet and promotes Th1-cell survival by regulating the proapoptotic gene Bim. Eur J Immunol. (2015) 45:1192–205. 10.1002/eji.201444633 25486906 PMC4406154

[22] Stittrich AB Haftmann C Sgouroudis E Kühl AA Hegazy AN Panse I. The microRNA miR-182 is induced by IL-2 and promotes clonal expansion of activated helper T lymphocytes. Nat Immunol. (2010) 11:1057–62. 10.1038/ni.1945 20935646

[23] Szabo SJ Kim ST Costa GL Zhang X Fathman CG Glimcher LH. A novel transcription factor, T-bet, directs Th1 lineage commitment. Cell. (2000) 100:655–69. 10.1016/S0092-8674(00)80702-3 10761931

[24] Lord GM Rao RM Choe H Sullivan BM Lichtman AH Luscinskas FW. T-bet is required for optimal proinflammatory CD4^+^ T-cell trafficking. Blood. (2005) 106:3432–9. 10.1182/blood-2005-04-1393 16014561 PMC1895048

[25] Barber DL Wherry EJ Masopust D Zhu B Allison JP Sharpe AH. Restoring function in exhausted CD8 T cells during chronic viral infection. Nature. (2006) 439:682–7. 10.1038/nature04444 16382236

[26] Scholzen T Gerdes J. The Ki-67 protein: From the known and the unknown. J Cell Phys. (2000) 182:311–322. 10.1002/(SICI)1097-4652(200003)182:3<311::AID-JCP1>3.0.CO;2-9 10653597

[27] Brode S Cooke A. Immune-potentiating effects of the chemotherapeutic drug cyclophosphamide. Crit Rev Immunol. (2008) 28:109–26. 10.1615/CritRevImmunol.v28.i2.20 18540827

[28] Linsley PS Brady W Urnes M Grosmaire LS Damle NK Ledbetter JA. CTLA-4 is a second receptor for the B cell activation antigen B7. J Exp Med. (1991) 174:561–9. 10.1084/jem.174.3.561 1714933 PMC2118936

[29] Förster R Schubel A Breitfeld D Kremmer E Renner-Müller I Wolf E. CCR7 coordinates the primary immune response by establishing functional microenvironments in secondary lymphoid organs. Cell. (1999) 99:23–33. 10.1016/S0092-8674(00)80059-8 10520991

[30] McKinstry KK Strutt TM Bautista B Zhang W Kuang Y Cooper AM. Effector CD4 T-cell transition to memory requires late cognate interactions that induce autocrine IL-2. Nat Commun. (2014) 5:1–12. 10.1038/ncomms6377 25369785 PMC4223689

[31] Link A Vogt TK Favre S Britschgi MR Acha-Orbea H Hinz B. Fibroblastic reticular cells in lymph nodes regulate the homeostasis of naive T cells. Nat Immunol. (2007) 8:1255–65. 10.1038/ni1513 17893676

[32] Britschgi MR Favre S Luther SA. CCL21 is sufficient to mediate DC migration, maturation and function in the absence of CCL19. Eur J Immunol. (2010) 40:1266–71. 10.1002/eji.200939921 20201039

[33] Kozai M Kubo Y Katakai T Kondo H Kiyonari H Schaeuble K. Essential role of CCL21 in establishment of central self-tolerance in T cells. J Exp Med. (2017) 214:1925–35. 10.1084/jem.20161864 28611158 PMC5502431

[34] Nakajima C Mukai T Yamaguchi N Morimoto Y Park WR Iwasaki M. Induction of the chemokine receptor CXCR3 on TCR-stimulated T cells: dependence on the release from persistent TCR-triggering and requirement for IFN-gamma stimulation. Eur J Immunol. (2002) 32:1792–801. 10.1002/1521-4141(200206)32:6<1792::AID-IMMU1792>3.0.CO;2-0 12115663

[35] Sasaki K Tsuji T Jinushi T Matsuzaki J Sato T Chamoto K. Differential regulation of VLA-2 expression on Th1 and Th2 cells: a novel marker for the classification of Th subsets. Int Immunol. (2003) 15:701–10. 10.1093/intimm/dxg066 12750354

[36] Ross SH Cantrell DA. Signaling and Function of Interleukin-2 in T Lymphocytes. Annu Rev Immunol. (2018) 36:411–33. 10.1146/annurev-immunol-042617-053352 29677473 PMC6472684

[37] Chang JT Palanivel VR Kinjyo I Schambach F Intlekofer AM Banerjee A. Asymmetric T lymphocyte adaptive immune responses. Science. (2007) 315:1687–92. 10.1126/science.1139393 17332376

[38] van Essen D Dullforce P Brocker T Gray D. Cellular interactions involved in Th cell memory. J Immunol. (2000) 165:3640–6. 10.4049/jimmunol.165.7.3640 11034367

[39] Linton PJ Harbertson J Bradley LM. A critical role for B cells in the development of memory CD4 cells. J Immunol. (2000) 165:5558–65. 10.4049/jimmunol.165.10.5558 11067910

[40] Whitmire JK Asano MS Kaech SM Sarkar S Hannum LG Shlomchik MJ. Requirement of B cells for generating CD4^+^ T cell memory. J Immunol. (2009) 182:1868–1876. 10.4049/jimmunol.0802501 19201839 PMC2658628

[41] Mollo SB Zajac AJ Harrington LE. Temporal requirements for B cells in the establishment of CD4 T cell memory. J Immunol. (2013) 191:6052–9. 10.4049/jimmunol.1302033 24218454 PMC3866023

[42] Dooms H Wolslegel K Lin P Abbas AK Interleukin-2 enhances CD4^+^ T cell memory by promoting the generation of IL-7Rα-expressing cells. J Exp Med. (2007) 204:547–57. 10.1084/jem.20062381 17312008 PMC2137906

