# Supplementary material for: Enhanced Cell Division Is Required for the Generation of Memory CD4 T Cells to Migrate Into Their Proper Location
Source: Front Immunol. 2020 Jan 15;10:3113. doi: 10.3389/fimmu.2019.03113 (PMC6974474; doi:10.3389/fimmu.2019.03113)

**Supplementary Material**

Figure S1. CXCR3 is an alternative marker for T-bet in activated CD4 T cells.

(A and B) C57BL/6 mice were transferred with purified Thy1.1^+^ T-bet-ZsGreen tg LCMV GP-specific CD4 T cells, immunized with LCMV-gp61 and LPS and analyzed on day 6 after immunization by flow cytometry.

(A) The expression of T-bet and CXCR3 in Thy1.1^+^CD4^+^B220^−^NK1.1^−^PI^−^ cells of the spleen and BM. n=3.

(B) Most CD49b^+^T-bet^+^ activated CD4 T cells express CXCR3. The dot plot displays the expression of CD49b and T-bet in activated CD4 T cells. A histogram shows CXCR3 expression in the CD49b^+^T-bet^+^ subpopulation (a dot line, isotype control). n=3.

(C) CXCR3^+^ activated CD4 T cells preferentially migrate into the BM. C57BL/6 mice were transferred with purified Thy1.1^+^ LCMV GP-specific CD4 T cells and immunized with LCMV-gp61 and LPS. Sorted Thy1.1^+^ cells from the spleen on day 6 after immunization were transferred into RAG1-deficient mice and 2 h later the mice were analyzed for Thy1.1^+^ cell numbers in the spleen and BM. The transfer efficiency was compensated by co-transferred Ly5.1^+^ splenocytes. Dot plots show the expression of CXCR3 in Thy1.1^+^CD44^hi^ CD4 T cells of the spleen before transfer (left) and of the spleen (middle) and BM (right) after transfer. n=4. Data is shown as mean ± SD (dot line: before transfer).

(D) CD49b^+^CXCR3^+^ memory precursors exhibit enhanced cell division. Purified CFSE-labeled Ly5.2^+^ ovalbumin-specific CD4 T cells were transferred intravenously into Ly5.1 C57BL/6 mice followed by intraperitoneal immunization with 100 µg ovalbumin and 10 µg LPS, and analyzed on day 5 after immunization by flow cytometry. The percentages of each CFSE-diluted population are shown. n=6.

(E) BM memory CD4 T cells express PD-1 lowly. C57BL/6 mice harboring LCMV GP-specific memory CD4 T cells were analyzed for the expression of PD-1 by flow cytometry on day 40 after immunization. A histogram shows PD-1 expression in splenic (dot line) and BM (solid line) memory CD4 T cells, and splenic CD44^hi^ CD4 T cells (filled). n=2.

(F) Injection of anti-IL-2 antibodies does not affect the expression of CCR7. As described in Fig. 6B, C57BL/6 mice were transferred, immunized and analyzed by flow cytometry on day 6 after immunization, receiving 1 mg of anti-IL-2 or isotype control on days 0, 2 and 4. The histogram shows the expression of CCR7 in Thy1.1^+^CD44^hi^CD49b^-^ CD4 T cells. The bar charts display the MFI of CCR7 expression. n=5.

These data are representative of two independent experiments. ***: p<0.001.


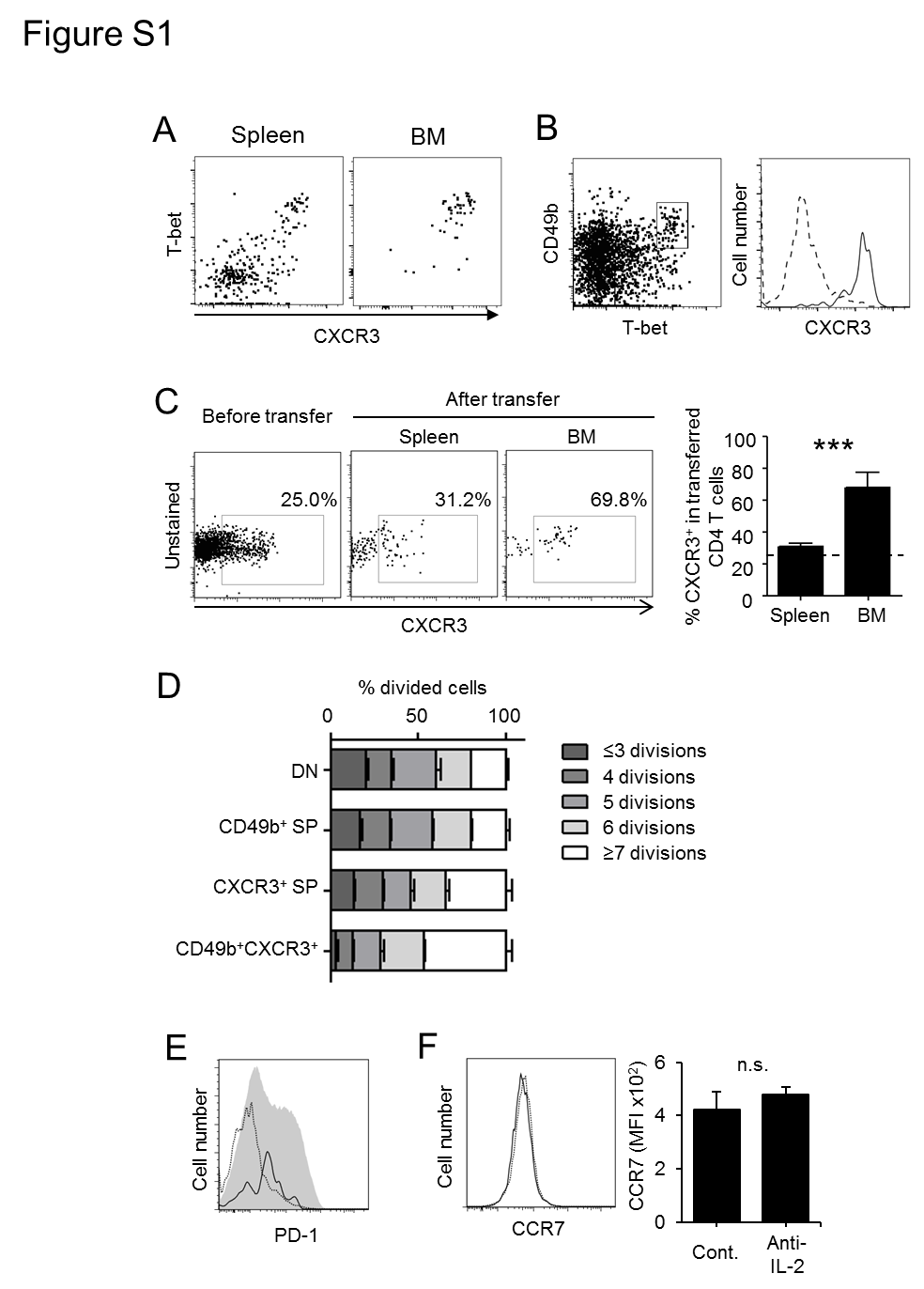

Supplement: Supplementary file 1 [file Data_Sheet_1.docx]
